# Convergence of the dimensional assessment of personality pathology (DAPP-BQ) and the five-factor personality inventory for the international classification of diseases 11th edition (FFiCD) in the context of the five-factor model and personality disorders

**DOI:** 10.1186/s12888-024-05835-8

**Published:** 2024-05-21

**Authors:** Anton Aluja, Ferran Balada, Kokou A. Atitsogbe, Jérôme Rossier, Luis F. García

**Affiliations:** 1 Human Behavior Laboratory, Lleida Institute for Biomedical Research Dr. Pifarré Foundation (IRBLleida), Catalonia, Spain; 2 University of Lleida, Catalonia, Spain; 3 Deparment of Psychobiology, Autonomous University of Barcelona, Catalonia, Spain; 4 Institute of Psychology, Faculty of Social and Political Sciences, University of Lausanne, Lausanne, Switzerland; 5 Department of Biological and Health Psychology, Autonomous University of Madrid, Madrid, Spain

**Keywords:** DAPP-90, FFiCD, NEO-FFI-R, Structural equation models, Personality disorders, ICD-11, APA

## Abstract

The current manuscript presents the convergence of the Dimensional Assessment of Personality Pathology (DAPP-BQ), using its short form the DAPP-90, and the Five-Factor Personality Inventory for International Classification of Diseases (ICD-11), the FFiCD, in the context of the five-factor personality model and the categorical approach of personality disorders (PDs). The current manuscript compares the predictive validity of both the FFiCD and the DAPP-90 regarding personality disorder scales and clusters. Results demonstrate a very high and meaningful convergence between the DAPP-90 and the FFiCD personality pathology models and a strong alignment with the FFM. The DAPP-90 and the FFiCD also present an almost identical predictive power of PDs. The DAPP-90 accounts for between 18% and 47%, and the FFiCD between 21% and 47% of PDs adjusted variance. It is concluded that both DAPP-90 and FFiCD questionnaires measure strongly similar pathological personality traits that could be described within the frame of the FFM. Additionally, both questionnaires predict a very similar percentage of the variance of personality disorders.

**Supplementary Information:**

The online version contains supplementary material available at 10.1186/s12888-024-05835-8.

## Introduction

Personality pathology refers to inflexible, stable, maladaptive patterns of emotions, cognitions, and behaviors generating distress and self and interpersonal functioning difficulties. A new approach to personality pathology was included in the Statistical Manual of Mental Disorders, Fifth Edition [1] (DSM-5), as an alternative approach to the categorical diagnosis of Personality Disorders (PDs). This Alternative Model of Personality Disorder (AMPD) proposed two fundamental criteria: The difficulty in the functioning of personality (Criterion A) and the configuration of five maladaptive personality traits (Criterion B). Criterion A estimates clinical prognosis and the intensity of the treatment, and Criterion B is designed to help determine the best approach to treatment [2]. This criterion B is measured with the Personality Inventory for DSM-5 (PID-5) [3], which contains 25 facets grouped into five domains (Negative affectivity, Detachment, Antagonism, Disinhibition, and Psychoticism). Criteria A and B represent severity and style of personality disorders, respectively [4].

In parallel to the AMPD, another dimensional model of PDs was developed within the framework of the 11th revision of the International Classification of Diseases (ICD-11) [5]. The ICD-11 presents two-stage evaluation with severity level as the primary stage and domain traits as the secondary one. In fact, whereas the severity/functioning criterion is central to diagnosis in the AMPD, it is the only necessary criterion in the ICD-11 [5]. The secondary level (trait specifiers) is considered optional and only aimed at describing the specific type of problems experienced by patients. This secondary level also consists of five pathological domains: Negative Affectivity, Detachment, Dissociality, Disinhibition, and Anankastia [6, 7]. To measure these pathological domains, Oltmanns and Widiger [8], developed the Personality Inventory for ICD-11 (PiCD). This instrument has proved to have both adequate internal consistency and a stable cross-cultural factor structure [9]. This structure, however, typically shows four rather than five factors, with Anankastia located at the opposite pole of Disinhibition [10, 11]. More recently, Oltmanns and Widiger [12], developed the Five-Factor Personality Inventory for the ICD-11 (FFiCD) to include facets in the measurement of those five PiCD domains. Given the time since their publication, the PiCD and, especially, the new FFiCD have received less empirical support than the PID-5.

Prior to the release of both the PID-5 and PiCD, some dimensional personality pathology models had already been proposed. One of them was developed by Livesley and colleagues [13, 14], who developed the Dimensional Assessment of Personality Pathology-Basic Questionnaire (DAPP-BQ) [15]. The DAPP-BQ includes 290 items that measure four domains (Emotional Dysregulation, Social Avoidance, Dissocial Behavior, and Compulsivity) through 18 facets. It has demonstrated a robust factor structure in different cultural contexts ([16–20], including Spain [21]). Regarding predictive validity, the four domains of the DAPP-BQ explained between 29% and 63% of the variance of categorical PDs [16]. Considering both the psychometric soundness of the DAPP-BQ and the practical disadvantages of its length in clinical settings, Aluja et al. [22] proposed a 90-item short version (DAPP-90). This short version had a total factor congruence coefficient of 0.98 with the full 290-item structure, and adequate alpha reliabilities ranging from 0.70 to 0.82.

Aluja et al. [23] correlated DAPP-90 and PID-5 scales, revealing a substantial overlap, especially with the Emotional Dysregulation scale of the DAPP-90. Linear regressions also showed that both instruments predicted categorical PDs similarly. In clinical samples, Gutiérrez et al. [8] also found a substantial overlap between the DAPP-BQ and the PID-5. However, as far as we know, there is no such corresponding study comparing the DAPP-BQ with the FFiCD. The DAPP-BQ has also shown a substantial alignment with the Five-Factor Model of Personality (FFM). Thus, Emotional Dysregulation, Social Avoidance, Dissocial Behavior, and Compulsivity resemble the normal personality traits of Neuroticism, Extroversion, Agreeableness, and Conscientiousness, respectively [23]. Further studies confirmed this substantial overlapping [24, 25]. As expected, according to the dimensional models of personality disorders both the PiCD [26] and the FFiCD [11, 27, 28] have also shown a substantial alignment with the FFM. Thus, Negative Affectivity shows a strong positive relationship with Neuroticism, whereas Detachment, Dissocial, and Disinhibition show strong negative correlations with Extraversion, Agreeableness, and Conscientiousness, respectively. Finally, Anankastia presents the lowest fit to the FFM, although it is clearly associated with high Conscientiousness.

The current study was designed with three aims. To our knowledge, there is no study analyzing the convergence between the DAPP-BQ and the FFiCD, so the first and most important aim is to test to what extent the two questionnaires (DAPP-90 and FFiCD) overlap in a general community sample. To achieve this aim, exploratory and confirmatory factor analyses will be conducted. To better interpret this expected overlapping, both instruments will be analyzed conjointly with a measure of the FFM. Based on the literature, a substantial alignment among the three instruments (DAPP-90, FFiCD, and NEO-FFI-R [NEO Five Factor Inventory-Revised]) is expected. It should be remarked that testing the validity of FFiCD is relevant since ICD-11 is used by all WHO member states for coding purposes, national statistics, legal matters, insurance reimbursement, and other applied individual and social aims.

The second aim is to compare the criterion validity of DAPP-90 and FFiCD. In this way, the predictive power of both questionnaires over the ten categorical PDs and the three clusters will be compared. Although the classification of PDs into three clusters is not empirically supported and is overly heterogenous, we have also analyzed them to allow comparisons with other studies, and to test to what extent FFiCD and DAPP-90 are associated with the general features of the three clusters: Cluster A (unusual and eccentric beliefs and social distance), Cluster B (dramatic, overly emotional or unpredictable thinking or behavior, impulsivity and lack of empathy), and Cluster C (anxious, fearful thinking or behavior) [1].

Previous evidence about the most relevant relationships between categorical PDs and PiCD suggests that Paranoid is related to Negative Affectivity and Dissociality, Schizoid and Schizotypal to Detachment, Clusters B PDs mainly to Dissociality, although Borderline would present its strongest relationships with Negative Affectivity, and that PDs characterized by Anxiety (Cluster C: Avoidant, Dependent and Obsessive-Compulsive) are mostly related to Negative Affectivity, and Avoidant also to Detachment, and Obsessive-Compulsive also to Anankastia [29]. The same pattern of relationships to the facet-based version of the PiCD (FFiCD) is expected, as well as with the counterpart scales of the DAPP-90 [16]. In addition, scores on scales of both instruments will be compared in participants with and without a self-reported history of some mental illness using a similar method to that used by Le Corff et al. [30] to test the differential construct validity.

Finally, and as a third and secondary aim, given the limited existing studies examining the psychometric characteristics of the newly introduced DAPP-90 and FFiCD, it remains imperative to verify whether the psychometric properties of both instruments, as reported in initial studies [12, 22, 23, 28, 30], are reproducible across diverse sample sets.

## Method

### Participants and procedure

A total of 504 unpaid community volunteers (259 women and 245 men) with a mean age of 45.05 (*SD* = 18.22) and 45.43 (*SD* = 17.62), respectively, participated in this study. There were no significant differences in age between genders, *t*(1) = 2.42, *p* = .81. The age range was between 18 and 92 years old (with the following percentages by age ranges: *≤* 30 = 25.4%; 31 and *≤* 40 = 16.13%; 41 and *≤* 50 = 16.5%; 51 and *≤* 60 = 19.6% and > 61 = 22.2%).

Participants were recruited from the general population by undergraduate students trained in the theory and instruments of FFM and dimensional models of PDs. As a regular exercise, they had to administer the protocol to six people: one male and female aged between 18 and 30 years, one male and female aged between 31 and 50 years, and one male and female older than 50 years. All participants were informed through written consent according to the study guidelines approved by the University ethical committee. A question about psychopathological status was included in the paper-pencil protocol asking whether the participant had been diagnosed with any of the following mental disorders: Alcoholism or drug addiction, anxiety-depression, attention-deficit hyperactivity, eating, obsessive compulsive, personality, or post-traumatic stress. A total of 65 participants (12.9%) reported some disorder. The great variety of conducted analyses will be described as results are reported.

### Instruments

#### The five-factor personality inventory for ICD-11 (FFiCD; [11])

The FFiCD Spanish validated version used in this study was adapted by Sorrel et al. [31]. This questionnaire includes 121 items assessing 5 maladaptive personality domains and 20 facets: Negative Affectivity (Anger, Anxiousness, Emotional Lability, Mistrustfulness, Depressiveness, Shame, and Vulnerability), Anankastia (Inflexibility, Perfectionism, and Workaholism), Dissociality (Aggression, Lack of Empathy, and Self-Centeredness), Disinhibition (Disorderliness, Irresponsibility, Rashness, and Thrill-Seeking), and Detachment (Emotional Detachment, Social Detachment, and Unassertiveness). Items are answered on a five-point Likert-type scale ranging from 1 (Strongly disagree) to 5 (Strongly agree). FFiCD factor structure and internal alpha consistency were satisfactory in both the original and Spanish adaptation studies ([12, 31], for details).

#### The Dimensional Assessment of Personality Pathology-Basic questionnaire – 90-item shortened version (DAPP-90; [21])

The DAPP-90 is a 90-item short form of the Dimensional Assessment of Personality Pathology-Basic Questionnaire (DAPP-BQ) ([15], proposed by Aluja et al. [22]), using structural equation modeling techniques to select the best items. This short version was authorized by the workgroup of the Spanish adaptation of the DAPP-BQ [21]. This self-report questionnaire uses a 5-point scale ranging from 1 (Very unlike me) to 5 (Very like me). Like the original long version, the DAPP-90 has 18 facets (Anxiety, Cognitive Distortion, Submissiveness, Identity Problems, Affective Instability, Oppositionality, Insecure Attachment, Suspiciousness, Low Affiliation, Intimacy Problems, Restricted Expression, Callousness, Conduct Problems, Stimulus Seeking, Rejection, Narcissism, Compulsivity, and Self-Harm) grouped into four factors: Emotional Dysregulation, Social Avoidance Dissocial Behavior, and Compulsiveness (see Aluja et al. [22], for details about the DAPP-90 Spanish shortened version).

#### NEO five factor inventory-revised (NEO-FFI-R; [32])

The NEO-FFI-R is a 60-item shortened version of the NEO-PI-R [33]. The NEO-FFI-R measures Neuroticism, Extraversion, Openness, Agreeableness, and Conscientiousness normal personality domains. Items are answered using a 5-point Likert scale. The Spanish version of the NEO-FFI-R was validated by Aluja et al. [34] and presented similar reliability coefficients to the NEO-FFI or the original English-version of the NEO-FFI-R versions.

#### International personality disorder examination (IPDE; [35])

The IPDE screening questionnaire is a self-administered questionnaire that contains 77 items. The participants respond either True or False to questions about the DSM-IV criterion of the categorical PDs: Paranoid, Schizoid, Schizotypal, Antisocial, Borderline, Histrionic, Narcissistic, Avoidant, Dependent, and Obsessive-Compulsive [35]. The internal consistency of these scales is usually low due to the heterogeneity of the DSM-IV PD symptoms and criteria [36]. The Spanish version [37], was used in the present study, and PDs and clusters were analyzed. PD Clusters A, B, and C were computed by adding the scale scores of the corresponding PDs: Cluster A: Paranoid, Schizoid, and Schizotypal; Cluster B: Antisocial, Borderline, Histrionic, and Narcissistic; and Cluster C: Avoidant, Dependent, and Obsessive-compulsive.

## Results

### Convergence between the DAPP-BQ and the FFiCD

#### Product-moment correlation analysis between FFiCD, DAPP-90, and NEO-FFI-R

Table 1 shows the correlation matrix between the FFiCD and DAPP-90, and both with the NEO-FFI-R. DAPP Emotional Dysregulation, Social Avoidance, and Dissocial Behavior correlate strongly with several domains of the FFiCD, especially with Negative Affectivity, Detachment, and Dissociality, respectively. Compulsivity shows a high correlation with Anankastia (0.58). Neuroticism from the NEO-FFI-R correlates highly with FFiCD Negative Affectivity (0.79), followed by Disinhibition (0.51) and Detachment (0.42). Extraversion correlates negatively with Detachment (-0.58) and with Negative Affectivity (-0.34). Openness correlates discreetly with Detachment only (-0.14). Agreeableness correlates strongly and negatively with all FFiCD domains but positively with Anankastia (0.11). Conscientiousness correlates negatively with four out five FFiCD domains, especially with Disinhibition (-0.65), and positively with Anankastia.

**Table 1:** Pearson correlation matrix between DAPP-90, FFiCD, and NEO-FFI-R

	Neuroticism	Extraversion	Openness	Agreeableness	Conscientiousness	Emotional Dysregulation	Dissocial Behavior	Social Avoidance	Compulsivity
*DAPP-90*									
Emotional Dysregulation	**.78** ^**1**^	− .30^**2**^	.01^2^	− .31^2^	**− .44** ^**1**^				
Dissocial Behavior	.30^3^	.07^3^	.10^2^	**− .55** ^1^	− .39^**1**^				
Social Avoidance	**.52** ^**2**^	**− .55** ^**1**^	− .16^1^	− .28^2^	− .35^**1**^				
Compulsivity	.13^4^	− .07^3^	− .01^2^	.10^3^	.36^**1**^				
*FFiCD*									
Negative Affectivity	**.79** ^**1**^	− .34^**2**^	.00^**1**^	− .35^**2**^	− .36^**2**^	**.87** ^**1**^	**.48** ^**3**^	**.61** ^**2**^	.16^**2**^
Detachment	**.42** ^**2**^	**− .58** ^**1**^	− .14^**1**^	− .31^**3**^	− .25^**3**^	**.46** ^**3**^	.16^**4**^	**.70** ^**1**^	.09^**2**^
Dissociality	.24^**3**^	− .04^**3**^	.01^**1**^	**− .66** ^**1**^	**− .43** ^**2**^	**.41** ^**3**^	**.70** ^**1**^	.29^**4**^	− .08^**2**^
Disinhibition	**.51** ^**2**^	− .15^**3**^	.07^**1**^	**− .47** ^**2**^	**− .65** ^**1**^	**.70** ^**2**^	**.60** ^**2**^	**.47** ^**3**^	− .16^**2**^
Anankastia	.14^**3**^	− .09^**3**^	− .03^**1**^	.11^**4**^	**.43** ^**2**^	.19^**4**^	− .01^**5**^	.16^**4**^	**.58** ^**1**^

Note: *r* equal to or higher than 0.10, *p* < .05; 0.12, *p* < .01; 0.15, *p* < .001. Correlations higher than ± 0.40 are in boldface. Correlations of Agreeableness with Negative Affectivity (-0.35) and Detachment (-0.31) are not significantly different. However, it has been decided to include a different superscript to remark that the difference between the latter and the correlation with Disinhibition (-0.47) is significant

Regarding the DAPP-90, Neuroticism correlates strongly with Emotional Dysregulation (0.78), and Social Avoidance (0.52). Correlations of Extraversion are negative with Social Avoidance (-0.55) and Emotional Dysregulation (-0.30). Openness correlates negatively but to a lower extent ​​with Social Avoidance (-0.16). Agreeableness correlates negatively with the first three domains of the DAPP-90 (Emotional Dysregulation, Dissocial Behavior, and Social Avoidance). Finally, Conscientiousness also correlates negatively with the first three domains of the DAPP-90, and positively with Compulsivity.

The interpretation of the relationships between DAPP-90 and FFiCD could be somewhat misleading since although two correlations may look different, that difference could not be significant. So, an exact calculus [see https://www.quantpsy.org/corrtest/corrtest2.htm [38] was used to test what correlations were significantly different within correlations of each pair of domains. We applied a one-tailed contrast and, considering the large sample size, a *p* lower than 0.01. It is important to note that all correlations were compared on its absolute value since we are testing if the magnitude of the relationships is different irrespective of the sign. As a result of this analysis, a superscript was added to the coefficients on Table 1. The highest correlation is always marked as the superscript 1. Different superscripts point out that those correlations are statistically different, with one exception commented below the table. Note that correlations were compared within a column only and separately for the DAPP-90 and the FFiCD. No comparison of correlations was conducted within a line.

#### Structural equation model of relationships between DAPP-90 and FFiCD

**Fig. 1:**
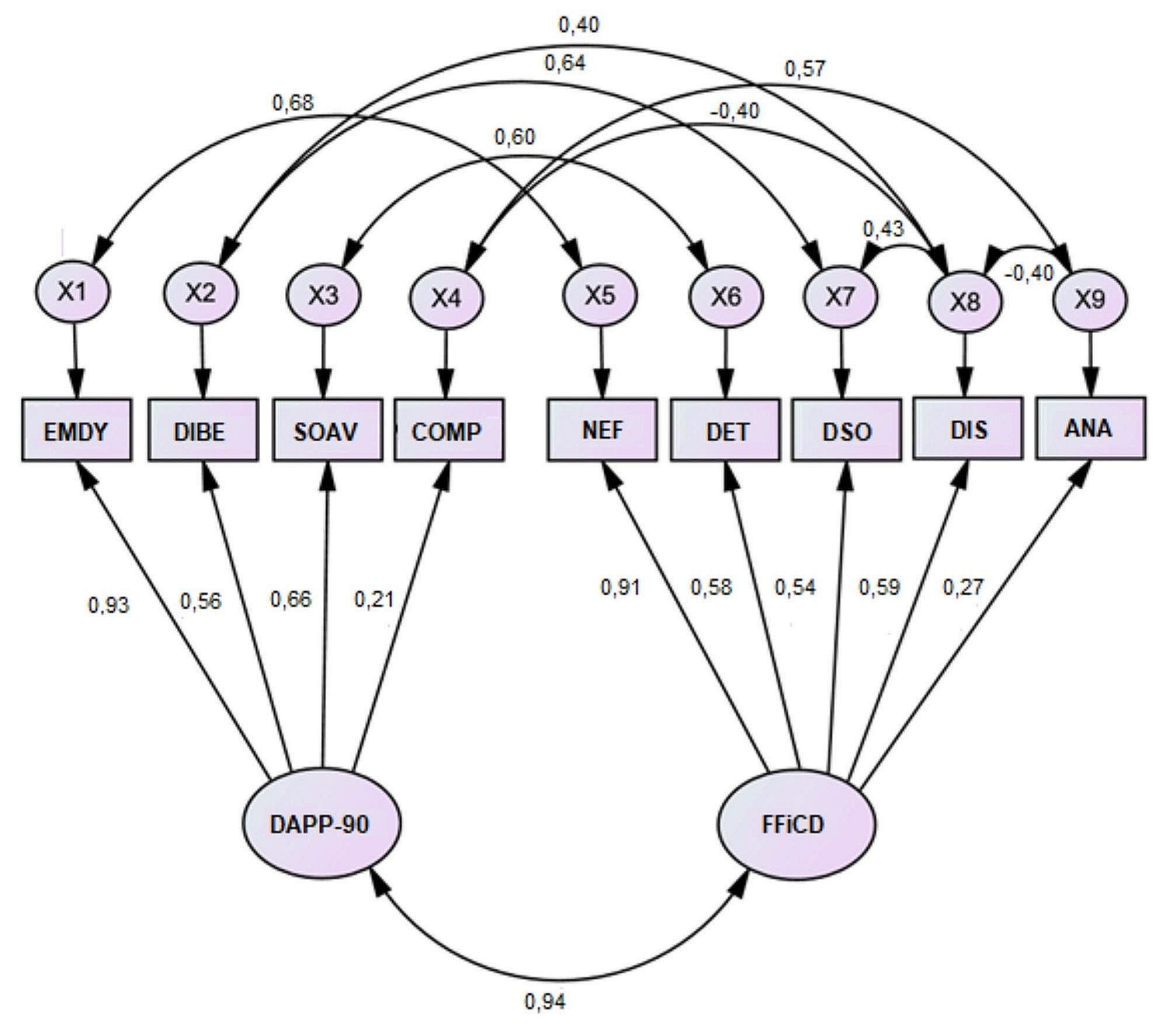
Path analysis diagram linking DAPP-90 and FFiCD structural models Note: EMDY: Emotional Dysregulation; DIBE: Dissocial Behavior, SOAV: Social Avoidance; and COMP: Compulsivity; NEF: Negative Affectivity; DET: Detachment; DSO: Dissociality; DIS: Disinhibition; ANA: Anankastia

Table 1 suggests a strong association between the DAPP-90 and FFiCD, so we have tested this hypothesis using structural equation modeling. Figure 1 shows the developed model. This model compares both questionnaires, including the domains as observed variables and defining a general latent factor for both questionnaires. Note that the correlation between both general factors was included as a parameter. The estimation method was Maximum likelihood due to the sample size and the normality of variables. To achieve the identification of the model, the error terms of observed variables were set to 1. Subsequently, the Modification Indices (MIs) were examined, and error terms of highly related facets were allowed to covary. This procedure has been used to compare constructs measured by questionnaires that have demonstrated high overlapping through correlations or exploratory factor analysis (EFA) [39, 40]. The following baseline comparisons of goodness-of-fit indices were used to assess the model Confirmatory Factor Analysis (CFA): Normed Fit Index (NFI), Relative Fit Index (RFI), Incremental Fit Index (IFI), Tucker-Lewis Index (TLI), and Comparative Fit Index (CFI). In general, a value higher than 0.90 on those indices is considered a good fit. In our model, values ​​of 0.95, 0.91, 0.96, 92, and 0.96 were observed, respectively, suggesting a proper fit of the model. The correlation between the two general factors of both instruments was 0.94.

### Criterion validity of DAPP-90 and FFiCD

#### Pearson correlation matrix of FFiCD, DAPP-90, and NEOFFI-R with IPDE personality clusters scores

Table 2 shows the correlations of the DAPP-90, FFiCD, and NEO-FFI-R with the IPDE personality disorders scales and the three clusters (A, B, and C). Regarding the FFiCD, it is observed that negative affectivity correlates with all PDs, especially with cluster A and C disorders. Disinhibition and Dissociality correlated mainly with cluster B disorders, as expected, Detachment mainly with Cluster A, and Anankastia mainly with Obsessive-Compulsive PD. Results are similar for the DAPP-90 scales. Emotional Dysregulation correlated with all clusters, Social Avoidance with Cluster A and C, and Dissocial Behavior with Cluster B. Compulsivity presents its largest correlation with Obsessive-Compulsive PD (0.30). Finally, the Neuroticism, Agreeableness, and Conscientiousness scales of the NEO-FFI-R showed significant correlations with all clusters of the IPDE. Extraversion also presented a relatively strong relationship with Cluster A. Openness barely correlated with any PD but presented some negative correlations with Cluster A (-0.17) and C (-0.14).

**Table 2:** Pearson correlation matrix between DAPP-90, FFiCD, NEO-FFI-R with IPDE personality disorders scores

	Paranoid	Schizoid	Schizotypal	Histrionic	Antisocial	Narcissistic	Borderline	Obsessive-Compu.	Dependent	Avoidant	CLUSTER A	CLUSTER B	CLUSTER C
*DAPP-90*													
Emotional Dysregulation	**.46** ^**1**^	.21^**2**^	**.49** ^**1**^	.33^**1**^	.27^**2**^	.21^**2**^	**.67** ^**1**^	.36^**1**^	**.51** ^**1**^	**.58** ^**1**^	**.50** ^**2**^	**.52** ^**2**^	**.63** ^**1**^
Dissocial Behavior	**.40** ^**1**^	− .05^**3**^	.32^**2**^	**.45** ^**1**^	**.51** ^**1**^	**.53** ^**1**^	**.43** ^**2**^	.21^**2**^	.24^**2**^	.25^**2**^	.29^**3**^	**.63** ^**1**^	.31^**3**^
Social Avoidance	**.40** ^**1**^	**.50** ^**1**^	**.57** ^**1**^	.01^**2**^	.17^**2**^	.09^**2**^	.36^**2**^	.20^**2**^	.32^**2**^	**.59** ^**1**^	**.63** ^**1**^	.22^**3**^	**.50** ^**2**^
Compulsivity	.03^**2**^	.10^**2**^	.02^**3**^	− .12^**2**^	− .14^**2**^	.02^**3**^	− .02^**3**^	.30^**1**^	.06^**3**^	.15^**2**^	.06^**4**^	− .08^**4**^	.18^**3**^
*FFiCD*													
Negative Affectivity	**.50** ^**1**^	.24^**2**^	**.51** ^**1**^	.28^**2**^	.28^**2**^	.20^**2**^	**.65** ^**1**^	.39^**1**^	**.46** ^**1**^	**.64** ^**1**^	**.54** ^**1**^	**.49** ^**2**^	**.65** ^**1**^
Detachment	.38^**1**^	**.50** ^**1**^	**.50** ^**1**^	− .01^**3**^	.16^**2**^	.08^**3**^	.31^**2**^	.23^**2**^	.25^**2**^	**.50** ^**2**^	**.59** ^**1**^	.19^**3**^	**.44** ^**2**^
Dissociality	**.42** ^**1**^	.10^**2**^	.36^**2**^	**.41** ^**1**^	**.54** ^**1**^	**.53** ^**1**^	**.41** ^**2**^	.16^**2**^	.20^**2**^	.23^**3**^	.38^**2**^	**.63** ^**1**^	.25^**3**^
Disinhibition	**.41** ^**1**^	.11^**2**^	**.44** ^**1**^	**.43** ^**1**^	**.48** ^**1**^	.29^**2**^	**.61** ^**1**^	.13^**2**^	.38^**1**^	.36^**3**^	**.42** ^**2**^	**.62** ^**1**^	.38^**2**^
Anankastia	.06^**2**^	.13^**2**^	.03^**3**^	− .13^**3**^	− .19^**2**^	.03^**3**^	.01^**3**^	**.42** ^**1**^	.05^**3**^	.19^**4**^	.09^**3**^	− .08^**3**^	.19^**3**^
*NEO-FFI-R*													
Neuroticism	**.44** ^**1**^	.26^**2**^	**.49** ^**1**^	.28^**1**^	.23^**2**^	.11^**3**^	**.64** ^**1**^	.39^**1**^	**.50** ^**1**^	**.58** ^**1**^	**.51** ^**1**^	**.45** ^**1**^	**.64** ^**1**^
Extraversion	− .24^**2**^	**− .48** ^**1**^	− .37^**1**^	.14^**2**^	− .02^**3**^	.06^**3**^	− .18^**3**^	− .16^**2**^	− .18^**3**^	**− .46** ^**2**^	**− .46** ^**1**^	− .01^**2**^	− .36^**2**^
Openness	− .08^**3**^	− .22^**2**^	− .11^**2**^	.03^**2**^	− .03^**3**^	.01^**3**^	.03^**4**^	− .10^**2**^	− .12^**3**^	− .10^**4**^	− .17^**3**^	.02^**2**^	− .14^**3**^
Agreeableness	**− .45** ^**1**^	− .18^**2**^	− .37^**1**^	− .27^**1**^	**− .51** ^**1**^	**− .45** ^**1**^	− .39^**2**^	− .18^**2**^	− .17^**3**^	− .25^**3**^	**− .43** ^**2**^	**− .54** ^**1**^	− .27^**2**^
Conscientiousness	− .36^**1**^	− .12^**2**^	**− .41** ^**1**^	− .34^**1**^	**− .43** ^**1**^	− .21^**2**^	**− .47** ^**2**^	− .01^**3**^	− .34^**2**^	− .29^**3**^	− .38^**2**^	**− .49** ^**1**^	− .28^**2**^

Note: *r* equal or higher 0.10, *p* < .05; r equal or higher 0.12, *p* < .01; r equal or higher 0.15, *p* < .001. Correlations higher than ± 0.40 are in boldface

As in Table 1, it has been decided to include different superscripts for some correlations that are not significant to mark differences with the highest correlation of the upper superscript. Those cases were: Dissocial Behavior and Compulsivity with Schizoid, Social Avoidance and Compulsivity with Narcissistic, Dissociality and Anankastia with Avoidant, Extraversion and Conscientiousness with Paranoid, Neuroticism and Conscientiousness with Narcissistic, Openness and Conscientiousness with Obsessive-Compulsive, and Openness with both Agreeableness and Conscientiousness with Cluster C

As expected, Table 2 shows that some DAPP-90 and FFiCD scales correlate more than others with categorical PDs and clusters. So, a superscript has been also added to the correlations of this table to point out what correlations are statistically different considering each pair of domains. We have applied the same procedure described for Table 1. This further analysis is advisable to detect what correlations on Table 2 could be considered different from a statistical point of view.

#### Factor analysis of FFiCD, DAPP-90, NEO-FFI-R domains and IPDE scales

Table 3 shows an Exploratory Factor Analysis with principal axis extraction and oblimin rotation, including the FFiCD, DAPP-90, NEO-FFI-R domains, and the ten IPDE PDs scales. Five factors were extracted. The first factor is a Neuroticism factor in which Emotional Dysregulation (DAPP-90) and Negative Affectivity (FFiCD) are grouped together with four PDs (Dependent, Borderline, Avoidant, and Histrionic). The second factor is formed by Agreeableness (in negative) and includes Dissociality (FFiCD), Dissocial Behavior (DAPP-90), and Disinhibition (FFiCD), along with IPDE Narcissistic, Antisocial, and Paranoid. The third factor grouped Extraversion (in negative) with Detachment (FFiCD), Social Avoidance (DAPP-90), and Schizoid and Schizotypal PDs. The fourth factor grouped Conscientiousness, Anankastia (FFiCD), Compulsivity (DAPP-90), and Obsessive-Compulsive PD. Only Openness loaded on the fifth factor, although some small secondary loadings such as Disinhibition (FFiCD) were also observed on this factor. *3.2.3. Multiple regression analysis predicting PDs and Clusters A, B, and C from DAPP-90 and FFiCD domains.*

**Table 3:** Exploratory factor analysis with oblimin rotation of DAPP-90, FFiCD, NEO-FFI-R domains and IPDE scales

	I	II	III	IV	V	h^2^
***Neuroticism*** **(NEO-FFI-R)**	**0.77**	0.08	0.35	0.08	0.19	0.77
Dependent (IPDE)	**0.77**	0.10	0.06	0.00	− 0.26	0.66
Borderline (IPDE)	**0.76**	0.36	0.12	− 0.06	0.03	0.73
Emotional Dysregulation (DAPP-90)	**0.74**	0.24	0.36	0.11	0.34	0.86
Negative Affectivity (FFiCD)	**0.68**	0.28	***0.44***	0.18	0.33	0.87
Avoidant (IPDE)	**0.60**	0.12	***0.48***	0.15	− 0.08	0.62
Histrionic (IPDE)	**0.53**	***0.43***	− 0.33	− 0.15	− 0.04	0.60
Dissociality (FFiCD)	0.13	**0.85**	0.16	− 0.05	0.18	0.80
Dissocial Behavior (DAPP-90)	0.27	**0.78**	− 0.02	0.03	0.27	0.76
***Agreeableness*** **(NEO-FFI-R)**	− 0.07	**− 0.77**	− 0.27	0.13	0.05	0.69
Narcissistic (IPDE)	0.13	**0.74**	− 0.13	0.15	− 0.15	0.63
Antisocial (IPDE)	0.26	**0.67**	0.02	− 0.23	− 0.13	0.58
Disinhibition (FFiCD)	***0.51***	**0.51**	0.26	− 0.28	0.37	0.80
Paranoid (IPDE)	***0.42***	**0.46**	0.31	0.05	− 0.16	0.51
Detachment (FFiCD)	0.20	0.18	**0.80**	0.10	0.05	0.72
Social Avoidance (DAPP-90)	0.33	0.14	**0.78**	0.05	0.10	0.75
***Extraversion*** **(NEO-FFI-R)**	− 0.15	0.10	**− 0.77**	0.04	0.13	0.65
Schizoid (IPDE)	0.03	0.03	**0.72**	0.07	− 0.28	0.60
Schizotypal (IPDE)	***0.48***	0.33	**0.49**	− 0.03	− 0.24	0.64
Anankastia (FFiCD)	0.05	− 0.05	0.15	**0.87**	0.06	0.78
Compulsivity (DAPP-90)	0.05	− 0.06	0.09	**0.78**	0.04	0.63
***Conscientiousness*** **(NEO-FFI-R)**	***− 0.40***	− 0.38	− 0.26	**0.60**	− 0.05	0.74
Obsessive-Compulsive (IPDE)	***0.45***	0.18	0.08	**0.54**	− 0.25	0.59
***Openness*** **(NEO-FFI-R)**	0.00	0.00	− 0.24	0.01	**0.66**	0.50

Note: Factor loadings equal to or higher than ± 0.40 in boldface. In italics, secondary loadings

Equal to or higher than ± 0.40

Table 4 displays the multiple linear regression analysis by considering the DAPP-90 and FFiCD domains as independent variables (in separate analyses) and the 10 PDs scales from IPDE as dependent variables using the stepwise method. A more stringent criterion of PIN (probability of *F*-to-enter) was used *p* < .001, as well as the usual criterion of POUT (probability of *F*-to remove) *p* < .10. The DAPP-90 domains included in the equation predict between 18% (Obsessive-compulsive) and 47% (Borderline) of the adjusted variance (30% average). The FFiCD domains predict between 21% (Dependent) and 47% (Borderline), and the average was also 30% of the adjusted variance. Table 4 shows only the scales of both questionnaires that were considered for the regression equation with the corresponding standardized beta coefficients.

**Table 4:** Linear multiple regressions of DAPP-90 and FFiCD domains predicting IPDE personality disorder scales (dependent variable) (Standardized beta coefficients between brackets)

DAPP-90				FFiCD		
IPDE Scales	*R*	*R* ^2 adjusted^	*Factors*	*R*	*R* ^2 adjusted^	*Factors*
Antisocial	0.52	0.27	+DIBE, -COMP	0.59	0.34	+DSO, +DIS, -ANA
Avoidant	0.65	0.42	+SOAV, +EMDY	0.65	0.43	+NEF, +DET
Borderline	0.68	0.47	+EMDY, -COMP	0.69	0.47	+NEF, +DIS
Dependent	0.51	0.25	+EMDY	0.46	0.21	+NEF
Histrionic	0.45	0.20	+DIBE	0.51	0.26	+DIS, -DET, +DSO
Narcissistic	0.53	0.28	+EMDY, +COMP	0.53	0.28	+DSO
Obsessive-C.	0.43	0.18	+EMDT, +COMP	0.51	0.26	+ANA, +NEF
Paranoid	0.53	0.28	+EMDY, +DIBE, +SOAV	0.55	0.29	+NEF, +DSO
Schizoid	0.53	0.27	+SOAV, -DIBE	0.50	0.25	+DET
Schizotypal	0.60	0.36	+SOAV, +DIBE	0.50	0.25	+DET
*Average*	*0.54*	*0.30*		*0.55*	*0.30*	

Note: *R*: Multiple correlations. *R*^2^: Adjusted R square. DAPP-90 domains: EMDY: Emotional Dysregulation; DIBE: Dissocial Behavior; SOAV: Social Avoidance: COMP: Compulsiveness. FFiCD domains: NEF: Negative Affectivity; DET: Detachment; DSO: Dissociality; DIS: Disinhibition; ANA: Anankastia

Table 5 presents a multiple regression analysis using the enter method, considering the three clusters of categorical personality disorders as dependent variables and the FFiCD and DAPP-90 domains as independent ones. In this case, we have chosen the enter method to consider the contribution of all scales. Both instruments were analyzed separately to compare the predictive power and achieve the second aim. It is highlighted that adjusted *R*^2^ were almost identical across the three clusters for both instruments, and the nature of the variables in the equations was similar (cluster A adjusted *R*^2^ were 0.42 in both cases; cluster B, 0.49 vs. 0.48; and cluster C, 0.45 vs. 0.43). Similarly, note that the three clusters present a congruent pattern of relationships for both FFiCD and DAPP-90: Cluster A (Detachment and Social Avoidance), Cluster B (Dissociality and Dissocial behavior), Cluster C (Negative Affectivity and Emotional dysregulation).

**Table 5:** Multiple regression analysis predicting Clusters A, B and C, comparing DAPP-90 and FFiCD domains as independent variables (Standardized Coefficients). Method enter

DAPP-90 domains									
	Cluster A (*R* = .65; *R*^2 adjusted^ =.42)			Cluster B (*R* = .69. *R*^2 adjusted^ =.48)			*Cluster C* (*R* = .66. *R*^2 adjusted^ =.43)		
	β	*t*	*p*	β	*t*	*p*	β	*t*	*p*
(Constant)		-5.219	< .001		-2.649	.008		-4.732	< .001
Emotional Dysregulation.	.135	2.783	.006	.361	7.882	< .001	.494	10.281	< .001
Dissocial Behavior	.102	2.586	.010	.478	12.818	< .001	.022	.572	.568
Social Avoidance	.524	12.161	< .001	− .092	-2.247	.025	.174	4.054	< .001
Compulsivity	− .031	− .902	.368	− .122	-3.709	< .001	.117	3.413	< .001
FFiCD domains									
	Cluster A (*R* = .65; *R*^2 adjusted^ =.42)			Cluster B (*R* = .70. *R*^2 adjusted^ =.49)			Cluster C (*R* = .67. *R*^2 adjusted^ =.45)		
	β	*t*	*p*	β	*t*	*p*	β	*t*	*p*
(Constant)		-3.396	< .001		-3.186	.002		-4.419	< .001
Anankastia	− .090	-2.254	.025	− .051	-1.350	.178	.099	2.543	.011
Dissociality	.140	3.170	.002	.394	9.511	< .001	− .042	− .977	.329
Disinhibition	− .067	-1.136	.257	.269	4.878	< .001	− .039	− .674	.500
Detachment	.425	10.197	< .001	− .155	-3.969	< .001	.097	2.383	.018
Negative Affectivity	.300	5.389	< .001	.225	4.312	< .001	.621	11.387	< .001

Note: *R* = Multiple correlations. *R*^2^ = Adjusted R square

A multiple regression analysis was also conducted to predict the three PD clusters using the facets of both FFiCD and DAPP-90 questionnaires as independent variables (Table S1 in supplementary material). In this case and considering also the large number of independent variables in this analysis compared with the previous one that used domains, the input PIN was fixed to a *p*-value less than 0.001 using the stepwise method again given the large number of facets. Once again, the predictive power (adjusted *R*^2^) of the facets of both questionnaires indicates a strong similarity for the FFiCD compared to DAPP-90: Cluster A (0.45 vs. 0.48), cluster B (0.49 vs. 0.52) and cluster C (0.44 vs. 0.45), respectively.

#### Mean comparison of groups with and without a reported psychopathological disorder, internal consistency, and descriptive statistics for all participants

Table 6 shows the descriptive statistics (mean and standard deviation) of both groups with and without a described psychopathological disorder. The *t*-test and Cohen’s *d* [41], statistics are reported, which measure the relative strength of the differences between the means of both groups. The group of participants who reported a mental disorder obtained significantly higher means in four of the five domains of the FFiCD: Negative Affectivity, Detachment, Disinhibition and, to a lesser extent, Anankastia (*p* = .046). Scores on the DAPP-90 were also significantly higher in the group with mental disorders (Emotional Dysregulation, Dissocial Behavior, Social Avoidance, and Compulsivity). On the other hand, in the NEO-FFI-R, the group with psychopathological disorders obtains significantly higher scores in Neuroticism and lower scores in Extraversion and, to a lesser extent, Conscientiousness (*p* = .04). In the case of the IPDE scales, participants with psychopathological disorders score significantly higher on Paranoid, Schizotypal, Borderline, Dependent, and Avoidant. Finally, the internal alpha consistencies were very good (> 0.79) for the FFiCD and the DAPP-90, acceptable for the NEO-FFI-R (between 0.68 and 0.83), and lower for the IPDE scales. Descriptive statistics for the DAPP-90 and the FFiCD by gender are shown in the supplementary material (S2 and S3).

**Table 6:** Mean comparison of groups with and without a reported psychopathological disorder, internal consistency, and descriptive statistics for all participants of DAPP-90, FFiCD, NEOFFI-R, and IPDE

	No disorder (*n* = 439)		Mental disorder (*n* = 65)					All participants				
	*M*	*SD*	*M*	*SD*	*t*-test	*p*	*d*	*M*	*SD*	*S*	*K*	α
DAPP-90												
Emotional Dysregulation	76.44	24.10	97.42	24.51	-6.54	0.001	− 0.87	79.14	25.13	0.38	-45	0.85
Dissocial Behavior	50.69	14.69	54.91	14.45	-2.6	0.031	− 0.29	51.23	14.71	0.46	− 0.24	0.89
Social Avoidance	33.60	9.19	38.18	9.36	-3.75	0.001	− 0.50	34.19	9.33	0.41	− 0.31	0.83
Compulsivity	15.03	4.69	16.89	4.43	-3.01	0.003	− 0.40	15.26	4.70	− 0.01	− 0.51	0.83
FFiCD												
Negative Affectivity	97.65	25.98	121.05	28.58	-6.68	0.001	− 0.88	100.67	27.45	0.29	− 0.34	0.95
Detachment	29.88	7.62	33.45	7.69	-3.57	0.001	− 0.47	30.34	7.71	0.37	0.23	0.79
Dissociality	43.45	11.66	45.23	11.15	0.1.6	0.248	− 0.15	43.68	11.60	0.74	0.34	0.85
Disinhibition	52.60	14.24	60.57	14.49	-4.20	0.001	− 0.56	53.63	14.51	0.36	− 0.28	0.90
Anankastia	67.54	11.66	70.65	12.07	-1.99	0.046	− 0.27	67.94	11.75	− 0.12	− 0.04	0.83
NEO-FFI-R												
Neuroticism	19.20	8.02	26.29	9.08	-6.53	0.001	− 0.87	20.12	8.50	0.31	− 0.25	0.82
Extraversion	27.30	7.02	24.12	7.39	3.38	0.001	0.45	26.89	7.14	− 0.20	0.00	0.74
Openness	27.40	6.78	27.94	7.61	− 0.58.	0.559	− 0.08	27.47	6.89	0.01	− 0.16	0.68
Agreeableness	33.56	6.06	33.20	5.08	0.45	0.649	0.06	33.51	5.94	− 0.73	0.49	0.74
Conscientiousness	34.85	7.75	32.77	7.92	2.02	0.044	0.27	34.59	7.79	− 0.46	− 0.35	0.83
IPDE												
Paranoid	1.83	1.44	2.51	1.53	-3.51	0.001	− 0.47	1.92	1.47	0.85	0.37	0.41
Schizoid	2.20	1.46	2.54	1.42	1.75	0.081	− 0.24	2.24	1.46	0.50	− 0.29	0.42
Schizotypal	1.60	1.60	2.14	1.89	-2.49	0.013	− 0.33	1.67	1.65	1.01	0.57	0.58
Histrionic	2.14	1.56	2.28	1.53	− 0.66	0.512	− 0.09	2.16	1.56	0.76	0.32	0.43
Antisocial	1.08	1.31	1.25	1.26	− 0.96	0.338	− 0.13	1.10	1.31	1.47	2.25	0.54
Narcissistic	2.29	1.55	2.57	1.62	− 0.350	0.178	− 0.18	2.33	1.56	0.78	0.35	0.50
Borderline	1.88	1.84	2.92	2.12	-4.17	0.001	− 0.55	2.02	1.91	0.85	− 0.03	0.67
Obsessive-Comp.	2.84	1.66	3.12	1.75	-1.28	0.291	− 0.17	2.88	1.68	0.35	− 0.26	0.58
Dependent	1.44	1.51	1.95	1.87	-2.50	0.013	− 0.33	1.50	1.57	1.08	0.70	0.62
Avoidant	2.74	1.97	3.77	2.28	-3.83	0.001	− 0.51	2.88	2.04	0.49	− 0.60	0.67

Note: *M* = mean; *SD* = Standard Deviation; *d* = Cohens’ *d*; *S* = Skewness; *K* = Kurtosis; α = Cronbach’s alpha

(Cohen’s d, 0.20 ≥ small, 0.50 ≥ medium, 0.80 ≥ large)

#### Replication of factor structures of FFiCD, DAPP-90, and with NEO-FFI-R

Regarding the third aim, to replicate the structure of the DAPP-90 and the FFiCD, the factor structures for both instruments are reported in the supplementary material using the same extraction and rotation methods (Tables S4 and S5, respectively). As mentioned in the introduction section, the literature largely supports a four-factor structure for both questionnaires, so a four-factor solution was extracted. Results confirmed the stability of the four-factor structures of both instruments across different samples.

Finally, two further factor analyses were conducted to link the FFM with the DAPP-90 and the FFiCD separately. These analyses sought to test the alignment of normal personality and pathological personality, removing a possible bias by introducing two pathological instruments in the factor analyses. Tables S6 and S7 in the supplementary material show both five-factor solutions. They supported the great alignment among normal and pathological personality in both instruments since the expected relationships between normal traits and pathological facets are generally found.

## Discussion

As a new facet-based instrument (FFiCD) was also developed to measure the dimensional personality pathology domains of the ICD-11, it was necessary to study the overl between the FFiCD and the DAPP-90, which also includes facets. Correlation and factor analysis largely confirmed that domains measured after the FFiCD are quite close to three of the four domains of the personality pathology model developed by Livesley and colleagues [15, 42]. Thus, Negative Affectivity, Detachment, and Dissociality are quite close to Emotional Dysregulation, Social Avoidance, and Dissocial Behavior, respectively. Disinhibition was also strongly linked to several DAPP-90 domains, and Anankastia shares common variance with compulsivity.

Additionally, a structural equation model confirmed this high convergence of both instruments assessing pathological personality, since our model exhibited an appropriate fit, with a correlation between both instruments of 0.94. This result also suggests that both questionnaires globally measure a general and the same construct of pathological or maladaptive personality. In recent years, research has focused on the nature of a general factor of PDs [43]. McCabe et al. [44], suggested that it would reflect general impairments like those considered in Criterion A of the PDs of the DSM-5. Although this assumption was not directly tested in the present manuscript, since no measure of Criterion A was included, the fact that Negative Affectivity and Emotional Dysregulation domains had extremely higher loadings (> 0.90) on general factors of the FFiCD and DAPP-90, respectively, would imply some agreement with this hypothesis [45]. The good fit of this structural model is congruent with the Hierarchical Taxonomy of Psychopathology [HiTOP; 46]. This structure raises a general higher-order factor underlying three pathological spectra of the HiTOP largely convergent with the four domains of personality disorders as follows: internalizing disorders with negative affectivity, thought disorder with psychoticism, and antagonistic and disinhibited with externalizing disorders [46].

The first aim was also to examine the possible overlap of both questionnaires with the FFM. Similarly, FFiCD and DAPP-90 domains showed a great alignment with the domains of the FFM. Thus, Negative Affectivity and Emotional Dysregulation could be understood as extreme variants of Neuroticism, Detachment, and Social Avoidance of Extroversion, Dissociality and Dissocial Behavior of Agreeableness [47]. On the other hand, Conscientiousness presents a large overlap with the Disinhibition domain of the FFiCD and a more complex relationship with the DAPP-90 domains. This result confirms that obsessive-compulsive disorder is closer to Neuroticism [48] than to Conscientiousness. Finally, and as has been generally reported in the literature about relationships between the FFM and personality disorders [49], Openness hardly plays any role in the differences observed in personality pathology. The results of the present paper, therefore, clearly support the hypothesis that normal personality models and theories can be used as a unifying framework to organize personality pathology and mental disorders and to develop a framework addressed at personalized treatment and improved efficacy in clinical settings [48, 49]. Thus, the knowledge of personality traits or dimensional personality disorders domains can help clinicians to understand better psychopathological symptoms and real negative consequences on people’s lives [29, 50]. For instance, low scores on detachment could help to understand social anxiety profiles and drug intake behaviors to reduce social anxiety or anger manifestations in social settings, among other maladaptive behaviors.

The second objective was examined through two analytic approaches. The first one was to compare mean differences in FFiCD and DAPP-90 domains between a group of people who reported mental disorders during the last year, as done in other studies [30], and another group that did not report such disorders. The first group obtained significantly higher means on four out of five FFiCD domains and all DAPP-90 domains. As expected, large effect sizes (> 0.80) were observed for Negative Affectivity and Emotional Dysregulation. Note that a similar effect size was observed for Neuroticism, in agreement with the great alignment with the FFM.

The other approach was to conduct a linear analysis to compare the predictive power of both instruments. Results clearly concluded that the predictive capacity is high and extremely similar, which once again supports the high overlapping demonstrated at the structural level. Therefore, the FFiCD presents a high criterion validity, and shows itself to be a useful instrument to explain the observed differences in maladaptive personality traits and behaviors [31]. As theoretically expected, Cluster A is predicted by Detachment and Negative Affectivity, but also by Anankastia and Dissociality. Cluster B by four out of five domains (Dissociality, Disinhibition, Negative Affectivity and Detachment), and Cluster C is mainly associated with Negative Affectivity. All these results demonstrate that significant trait domains of the FFiCD largely cover the personality features of the three clusters [6]: Detachment and Cluster A (emotional and interpersonal distance, and isolation), Dissociality and Cluster B (disregard for social obligations and conventions and the rights and feelings of others), and Negative Affectivity and Cluster C (tendency to manifest a broad range of distressing emotions). The same conclusions could be drawn about DAPP-90 since the three clusters are mostly associated with the DAPP-90 counterparts scales: Cluster A (Social Avoidance), Cluster B (Dissocial behavior and Emotional Dysregulation), and Cluster C (Emotional Dysregulation). As a final statement, it should be noted that the present study largely supports that FFiCD and DAPP-90 capture stylistic features of PDs, but not directly the presence of a diagnosis, since this specific criterion has not been considered.

Finally, the third aim of this study was to replicate the psychometric properties of the FFiCD and DAPP-90. The results show a robust factor structure and high factor congruence (> 0.90) with the original American and Spanish matrices [12, 28]. The internal consistencies of the two questionnaires’ domains have also been satisfactory in different samples, and therefore, the present study replicated the good psychometric properties of both instruments, which was especially necessary for the FFiCD.

This study has some limitations, the most obvious one being the nature of the sample. Although information about past mental disorders was gathered, it was not a clinical sample. Psychometric properties and predictive validity of the FFiCD should be further analyzed in clinical samples. It would also be necessary to test the differences among people with and without disorders based on a diagnosis made by specialized clinicians and not based on self-report as in the present study. Thus, future studies should include clinical samples and people from the general population. It would also be convenient to develop cross-cultural studies since most available evidence about the FFiCD stems exclusively from American and Spanish cultural contexts, in other words, in two Western countries [12, 28, 31]. Another limitation is that no statistical test to compare standardized beta coefficients in the regression analysis was conducted. It poses questions regarding discriminant validity since cast some doubts about which DAPP-90 and FFiCD domains are more important domains in the prediction of a given PD or cluster.

In conclusion, the present study confirms the expected large overlap between the FFiCD, personality pathology (DAPP-90), and normal personality, considered here by means of the FFM. This overlap was found at the scale and structural levels. Compared with the DAPP-90, the FFiCD domains assesses very similar constructs (Negative Affectivity and Emotional Dysregulation; Detachment and Social Avoidance; Dissociality and Dissocial Behavior), and the high correlation between the general factors of both questionnaires indicates that they measure the same construct of pathological personality. Moreover, the FFiCD was as good as the other two models at predicting the variability in categorical personality disorders, reinforcing its usefulness in clinical contexts. Finally, the consistently replicated strong psychometric properties of the FFiCD and DAPP-90 further underscore their usefulness in practical settings.

### Electronic supplementary material

Below is the link to the electronic supplementary material.

Supplementary Material 1

## Data Availability

The datasets analyzed in this article are not publicly available. Requests to access the datasets should be directed to firts author (AA, anton.aluja@udl.cat).
