# Supplementary material for: Convergence of the dimensional assessment of personality pathology (DAPP-BQ) and the five-factor personality inventory for the international classification of diseases 11th edition (FFiCD) in the context of the five-factor model and personality disorders
Source: BMC Psychiatry. 2024 May 21;24:386. doi: 10.1186/s12888-024-05835-8 (PMC11110343; doi:10.1186/s12888-024-05835-8)
Supplement: Supplementary file 1 — Supplementary Material 1 [file 12888_2024_5835_MOESM1_ESM.docx]

SUPPLEMENTARY MATERIAL

S1. Multiple stepwise regression analysis predicting Cluster A, B and C, comparing DAPP-90 and FFiCD facets as independent variables.

(Standardized Coefficients).

| Cluster A | | | | Cluster B | | | | Cluster C | | | |
| --- | --- | --- | --- | --- | --- | --- | --- | --- | --- | --- | --- |
| DAPP-90 facets | | | | | | | | | | | |
| *R* = .70; *R*^2 adjusted =^ .48 | | | | *R* = .73; *R*^2 adjusted^ = .52 | | | | *R* = .67; *R*^2 adjusted^ = .45 | | | |
|  | Beta | *t* | *p* |  | Beta | *t* | *p* |  | Beta | *t* | *p* |
| (Constant) |  | -6.93 | .001 | (Constant) |  | -2.91 | .004 | (Constant) |  | -3.73 | .001 |
| Suspiciousness | .28 | 7.43 | .001 | Callousness | .27 | 7.37 | .001 | Submissiveness | .28 | 6.21 | .001 |
| Low affiliation | .21 | 5.33 | .001 | Affective instability | .30 | 7.47 | .001 | Suspiciousness | .17 | 4.50 | .001 |
| Intimacy problems | .14 | 4.22 | .001 | Stimulus seeking | .13 | 3.70 | .001 | Low affiliation | .20 | 4.72 | .001 |
| Restricted expression | .19 | 5.16 | .001 | Narcissism | .20 | 5.49 | .001 | Insecure attachment | .18 | 4.34 | .001 |
| Self-harm | .13 | 3.60 | .001 | Self-harm | .17 | 4.90 | .001 |  |  |  |  |
|  |  |  |  | Compulsivity | -.11 | -3.48 | .001 |  |  |  |  |
|  |  |  |  | Low affiliation | -.12 | -3.35 | .001 |  |  |  |  |
| FFiCD facets | | | | | | | | | | | |
| *R* = .67; *R*^2 adjusted =^ .45 | | | | *R* = .71; *R*^2 adjusted^ = .49 | | | | *R* = .66; *R*^2 adjusted^= .44 | | | |
|  | Beta | *t* | *p* |  | Beta | *t* | *p* |  | Beta | *t* | *p* |
| (Constant) |  | -5.33 | .001 | (Constant) |  | -8.94 | .001 | Anxiousness | .26 | 4.77 | .001 |
| Emotional Detachment | .39 | 9.88 | .001 | Self-Centeredness | .26 | 6.52 | .001 | Shame | .20 | 4.38 | .001 |
| Depressiveness | .23 | 5.54 | .001 | Rashness | .14 | 3.33 | .001 | Depressiveness | .23 | 4.45 | .000 |
| Mistrustfulness | .18 | 4.60 | .001 | Aggression | .16 | 3.60 | .001 | Perfectionism | .11 | 3.37 | .001 |
|  |  |  |  | Emotional Lability | .19 | 4.59 | .001 |  |  |  |  |
|  |  |  |  | Thrill-Seeking | .16 | 4.03 | .001 |  |  |  |  |

Note: R: Multiple correlations. R^2^: Adjusted R square.

S2. Gender comparison and internal consistency of DAPP-90 facets.

|  | Women | | Men | |  |  |  |  |
| --- | --- | --- | --- | --- | --- | --- | --- | --- |
|  | *M* | *SD* | *M* | *SD* | *t*-test | *p <* | *d* | α |
| Anxiety | 11.74 | 4.40 | 13.22 | 5.08 | -3.50 | .001 | -.31 | .80 |
| Cognitive distortion | 10.54 | 4.31 | 11.41 | 4.48 | -2.23 | .026 | -.20 | .79 |
| Submissiveness | 9.95 | 3.90 | 11.43 | 4.06 | -4.17 | .000 | -.37 | .80 |
| Identity problems | 10.33 | 4.11 | 10.95 | 4.61 | -1.60 | .111 | -.14 | .81 |
| Affective instability | 10.95 | 4.74 | 12.39 | 5.10 | -3.27 | .001 | -.29 | .87 |
| Oppositionality | 10.51 | 4.30 | 11.03 | 4.48 | -1.33 | .184 | -.12 | .82 |
| Insecure attachment | 11.64 | 4.12 | 12.02 | 4.12 | -1.03 | .303 | -.09 | .75 |
| Suspiciousness | 9.67 | 3.94 | 9.48 | 4.07 | 0.55 | .586 | .05 | .82 |
| Low affiliation | 10.78 | 4.32 | 11.27 | 4.64 | -1.21 | .228 | -.11 | .83 |
| Intimacy problems | 9.71 | 3.16 | 10.81 | 3.36 | -3.76 | .000 | -.34 | .61 |
| Restricted expression | 13.29 | 4.54 | 12.49 | 4.30 | 2.04 | .042 | .18 | .79 |
| Callousness | 9.16 | 3.51 | 8.16 | 2.94 | 3.46 | .001 | .31 | .71 |
| Conduct problems | 9.74 | 4.11 | 8.42 | 3.51 | 3.88 | .000 | .35 | .70 |
| Stimulus seeking | 10.37 | 4.42 | 8.59 | 4.02 | 4.72 | .000 | .42 | .83 |
| Rejection | 12.05 | 4.12 | 11.12 | 4.03 | 2.58 | .010 | .23 | .75 |
| Narcissism | 12.07 | 4.13 | 12.90 | 4.09 | -2.27 | .024 | -.20 | .77 |
| Compulsivity | 14.73 | 4.49 | 15.78 | 4.84 | -2.51 | .012 | -.22 | .83 |
| Self-Harm | 6.56 | 3.06 | 6.94 | 3.52 | -1.29 | .199 | -.11 | .88 |
| Emotional Dysregulation. | 75.65 | 24.11 | 82.45 | 25.67 | -3.06 | .002 | -.27 | .85 |
| Dissocial Behavior | 53.39 | 15.61 | 49.19 | 13.53 | 3.23 | .001 | .29 | .89 |
| Inhibition/Avoidance | 33.79 | 9.28 | 34.56 | 9.39 | -0.93 | .354 | -.08 | .83 |
| Compulsivity | 14.73 | 4.49 | 15.78 | 4.84 | -2.51 | .012 | -.22 | .83 |

*Note.* *M* = Mean; *SD* = Standard deviation; *d* = Cohen’s *d* (1988); α = Cronbach’s alpha.

S3. Gender comparison and internal consistency of FFiCD facets.

|  | Women | | Men | |  |  |  |  |
| --- | --- | --- | --- | --- | --- | --- | --- | --- |
|  | *M* | *SD* | *M* | *SD* | *t*-test | *p* < | *d* | α |
| Vulnerability | 14.11 | 4.31 | 11.76 | 4.22 | -6.17 | .000 | -.55 | .75 |
| Anxiousness | 16.62 | 5.07 | 15.27 | 4.94 | -3.03 | .003 | -.27 | .75 |
| Shame | 17.75 | 4.49 | 15.97 | 4.29 | -4.56 | .000 | -.41 | .68 |
| Depressiveness | 17.65 | 6.65 | 16.09 | 5.86 | -2.79 | .005 | -.25 | .85 |
| Emotional lability | 15.34 | 5.59 | 13.94 | 4.80 | -3.00 | .003 | -.27 | .85 |
| Anger | 17.06 | 5.16 | 15.79 | 4.77 | -2.87 | .004 | -.26 | .79 |
| Mistrustfulness | 6.78 | 2.63 | 6.96 | 2.57 | 0.75 | .451 | .07 | .67 |
| Perfectionism | 18.68 | 4.25 | 18.34 | 4.27 | -0.89 | .376 | -.08 | .67 |
| Workaholism | 17.39 | 4.18 | 17.31 | 4.23 | -0.21 | .832 | -.02 | .64 |
| Inflexibility | 32.66 | 5.74 | 31.44 | 5.84 | -2.37 | .018 | -.21 | .70 |
| Irresponsibility | 20.47 | 6.44 | 20.57 | 7.02 | 0.17 | .867 | .01 | .84 |
| Disorderliness | 10.96 | 4.05 | 11.22 | 3.90 | 0.75 | .452 | .07 | .74 |
| Thrill-Seeking | 6.36 | 2.51 | 7.19 | 2.71 | 3.59 | .000 | .32 | .64 |
| Rashness | 15.46 | 4.51 | 15.05 | 4.51 | -1.03 | .303 | -.09 | .74 |
| Lack of Empathy | 15.20 | 4.44 | 16.51 | 4.71 | 3.21 | .001 | .29 | .68 |
| Aggression | 11.10 | 4.01 | 12.93 | 4.40 | 4.87 | .000 | .43 | .68 |
| Self-Centeredness | 15.35 | 4.19 | 16.38 | 4.74 | 2.58 | .010 | .23 | .67 |
| Unassertiveness | 8.69 | 2.25 | 8.03 | 2.16 | -3.35 | .001 | -.30 | .45 |
| Emotional Detachment | 11.18 | 4.10 | 11.87 | 3.98 | 1.90 | .058 | .17 | .71 |
| Social Detachment | 10.03 | 3.53 | 10.92 | 3.59 | 2.81 | .005 | .25 | .75 |
| Negative Affectivity | 105.31 | 28.33 | 95.77 | 25.64 | -3.96 | .000 | -.35 | .95 |
| Detachment | 29.90 | 7.72 | 30.81 | 7.70 | 1.33 | .183 | .12 | .79 |
| Dissociality | 41.66 | 10.75 | 45.82 | 12.10 | 4.09 | .000 | .36 | .85 |
| Disinhibition | 53.25 | 14.14 | 54.04 | 14.90 | 0.61 | .542 | .05 | .90 |
| Anankastia | 68.73 | 11.51 | 67.09 | 11.97 | -1.57 | .117 | -.14 | .83 |

*Note.* *M* = Mean; *SD* = Standard deviation; *d* = Cohen’s *d* (1988). α = Cronbach’s alpha.

S4. Principal Component Analysis with direct oblimin rotation of DAPP-90 facets excluding Self-Harm facet (structure matrix), communalities and congruency coefficients (*CC*) with original factor structure (Aluja et al., 2018).

| DAPP-90 | **I** | II | III | IV | *h^2^* | *CC* |
| --- | --- | --- | --- | --- | --- | --- |
| Anxiety | **.86** | .27 | -.24 | .21 | .74 | *1* |
| Cognitive distortion | **.80** | .33 | -.28 | .09 | .65 | *.99* |
| Submissiveness_ | **.77** | .07 | -.37 | .19 | .66 | *1* |
| Identity problems | **.86** | .32 | **-.43** | .05 | .78 | *1* |
| Affective instability | **.83** | .38 | -.15 | .12 | .72 | *.99* |
| Low affiliation | **.69** | .10 | **-.60** | .17 | .67 | *.98* |
| Oppositionality | **.74** | **.45** | -.30 | -.14 | .67 | *1* |
| Insecure attachment | **.71** | .21 | -.12 | .31 | .54 | *.98* |
| Suspiciousness | **.63** | **.51** | -.39 | .21 | .56 | *.99* |
| Callousness | .31 | **.80** | -.23 | .06 | .68 | *.99* |
| Conduct problems | .34 | **.81** | -.12 | -.17 | .70 | *.98* |
| Stimulus seeking | .21 | **.77** | -.03 | -.17 | .63 | *.98* |
| Rejection | .33 | **.74** | .22 | .33 | .72 | *.98* |
| Narcissism | **.65** | **.52** | .09 | .35 | .65 | *1* |
| Intimacy problems | .27 | .05 | **-.69** | -.14 | .49 | *.93* |
| Restricted expression | .37 | .24 | **-.75** | .25 | .68 | *.99* |
| Compulsivity | .17 | -.09 | -.04 | **.89** | .81 | *1* |
| *Congruency coefficients* | *1* | *.99* | *.98* | *.96* |  | ***.99*** |

Note: I: Emotional Dysregulation, II: Dissocial Behavior, III Social Avoidance and IV: Compulsivity

CC: Congruence Coefficients. Loadings higher than ±.40 are shown in boldface.

S5- Principal Component Analysis with direct oblimin rotation of FFiCD facets (Structure matrix), communalities and congruency coefficients (*CC*) with original factor structure from Oltmanns and Widiger (2020b) study 3, provided by the authors.

| FFiCD | I | II | III | IV | *h^2^* | *CC* |
| --- | --- | --- | --- | --- | --- | --- |
| Vulnerability | **.85** | .02 | .09 | **.47** | .77 | *.93* |
| Anxiousness | **.86** | .16 | .22 | **.47** | .76 | *.88* |
| Shame | **.76** | .28 | .12 | .37 | .65 | *.94* |
| Depressiveness | **.84** | .02 | .20 | **.55** | .76 | *.96* |
| Emotional lability | **.84** | .01 | **.42** | .32 | .73 | *.98* |
| Anger | **.78** | .25 | **.45** | .22 | .74 | *.99* |
| Mistrustfulness | **.62** | .19 | **.46** | **.44** | .56 | *1* |
| Perfectionism | .18 | **.81** | -.06 | .11 | .68 | *.98* |
| Workaholism | .07 | **.79** | .07 | .08 | .64 | *.94* |
| Inflexibility | .21 | **.72** | -.26 | .36 | .67 | *.99* |
| Irresponsibility | **.75** | -.33 | **.42** | **.48** | .77 | *.98* |
| Disorderliness | **.56** | **-.43** | **.45** | .38 | .62 | *.99* |
| Thrill-Seeking | .30 | -.28 | **.73** | .02 | .59 | *.92* |
| Rashness | .**70** | -.14 | **.48** | .06 | .64 | *.99* |
| Lack of Empathy | .35 | -.05 | **.81** | .29 | .72 | *.86* |
| Aggression | **.41** | -.12 | **.83** | .10 | .72 | *.93* |
| Self-Centeredness | **.41** | .03 | **.76** | .33 | .67 | *.80* |
| Unassertiveness | .38 | -.04 | -.31 | **.57** | .54 | *.94* |
| Emotional Detachment | **.44** | .03 | .32 | **.82** | .75 | *.87* |
| Social Detachment | .35 | .18 | .14 | **.77** | .62 | *.87* |
| *Congruency coefficients* | *.93* | *.96* | *.92* | *.92* |  | *.93* |

*Note*. I: Negative Affectivity; II: Anankastia+/Disinhibition-; III: Dissociality. IV: Detachment.

CC: Congruence Coefficients. Loadings higher than ±.40 are in boldface.

S6. Principal component analysis and orthogonal rotation of DAPP-90 facets (Excluding Self-harm) and NEO-FFI-R dimensions.

|  | I | III | II | IV | V | *h^2^* |
| --- | --- | --- | --- | --- | --- | --- |
| Anxiety | **.83** | .18 | .13 | .04 | .09 | .75 |
| Cognitive distortion | **.73** | .23 | .21 | -.06 | .16 | .67 |
| Submissiveness | **.76** | .24 | -.05 | .03 | -.09 | .65 |
| Identity problems | **.80** | .29 | .19 | -.12 | -.05 | .77 |
| Affective instability | **.80** | .08 | .25 | -.05 | .07 | .72 |
| Oppositionality | **.66** | .14 | .33 | -.36 | .05 | .70 |
| Insecure attachment | **.71** | -.04 | .11 | .13 | -.27 | .62 |
| Suspiciousness | **.52** | .25 | ***.45*** | .07 | -.16 | .57 |
| Low affiliation | **.57** | .**64** | .04 | .01 | -.03 | .74 |
| Intimacy problems | .25 | .30 | .02 | -.30 | -.36 | .38 |
| Restricted expression | .20 | **.74** | .26 | .13 | -.04 | .67 |
| Callousness | .16 | .06 | **.80** | -.03 | -.21 | .72 |
| Conduct problems | .17 | .08 | **.76** | -.21 | .14 | .68 |
| Stimulus seeking | .07 | -.02 | **.72** | -.15 | .35 | .67 |
| Rejection | .27 | -.24 | **.70** | .25 | -.01 | .68 |
| Narcissism | **.65** | -.18 | **.40** | .20 | .00 | .65 |
| Compulsivity | .19 | .18 | -.04 | **.80** | .00 | .72 |
| Neuroticism | **.81** | .24 | .05 | -.12 | -.02 | .72 |
| Extraversion | -.20 | **-.75** | .12 | .09 | .18 | .66 |
| Openness | .07 | -.13 | .00 | .00 | **.86** | .76 |
| Agreeableness | -.10 | -.22 | **-.71** | .24 | .14 | .64 |
| Conscientiousness | -.31 | -.21 | -.37 | **.71** | .05 | .77 |

Note: Factor loadings equal or higher .40 in boldface. In italics secondary loadings equal or higher than ±.40.

I: 'Emotional Dysregulation; II: Social Avoidance; III: Dissocial Behavior; IV Compulsivity.

S7. Principal component analysis and ortoghonal rotation of FFiCD facets and NEO-FFI-R dimensions.

|  | I | II | III | IV | V | *h^2^* |
| --- | --- | --- | --- | --- | --- | --- |
| Vulnerability | **.85** | .00 | .03 | .23 | .11 | .78 |
| Anxiousness | **.79** | .14 | .19 | .27 | .07 | .76 |
| Shame | **.73** | .25 | .09 | .16 | -.03 | .64 |
| Depressiveness | **.79** | .00 | .17 | .34 | .08 | .77 |
| Emotional lability | **.77** | -.01 | .36 | .07 | -.05 | .73 |
| Anger | **.71** | .23 | **.40** | -.01 | -.13 | .74 |
| Mistrustfulness | **.51** | .21 | **.45** | .19 | -.04 | .55 |
| Perfectionism | .17 | **.78** | -.02 | .04 | .06 | .65 |
| Workaholism | .04 | **.79** | .10 | -.01 | -.03 | .64 |
| Inflexibility | .21 | **.69** | -.19 | .29 | -.05 | .65 |
| Irresponsibility | **.65** | -.33 | .38 | .26 | .10 | .76 |
| Disorderliness | **.45** | **-.43** | **.42** | .19 | .11 | .61 |
| Thrill-Seeking | .15 | -.22 | **.68** | -.12 | .32 | .66 |
| Rashness | **.65** | -.13 | .39 | -.19 | -.02 | .62 |
| Lack of Empathy | .15 | -.01 | **.81** | .09 | -.06 | .70 |
| Aggression | .22 | -.08 | **.80** | -.04 | .14 | .71 |
| Self-Centeredness | .23 | .05 | **.78** | .09 | -.08 | .67 |
| Unassertiveness | **.41** | -.04 | -.27 | **.43** | -.10 | .43 |
| Emotional Detachment | .29 | .03 | .39 | **.66** | -.17 | .69 |
| Social Detachment | .18 | .19 | .22 | **.77** | .14 | .73 |
| Neuroticism | **.83** | -.02 | .06 | .17 | -.04 | .72 |
| Extraversion | -.21 | .07 | .06 | **-.78** | .17 | .69 |
| Openness | .02 | .01 | -.01 | -.12 | **.93** | .88 |
| Agreeableness | -.12 | .13 | **-.76** | -.18 | .12 | .65 |
| Conscientiousness | -.34 | **.65** | -.38 | -.20 | .06 | .73 |

Note: Factor loadings equal or higher than ±.40 in boldface.

In italics secondary loadings equal or higher than ±.40
